# Post-embolization dynamic changes in Obsidio Conformable Embolic: volume and radiodensity analysis

**DOI:** 10.1186/s42155-026-00792-7

**Published:** 2026-10-24

**Authors:** Mohamad Harraka, Radhika Amin, Amir Pourmorteza, Emil I. Cohen, Bahman Afsari, Saher S. Sabri, Filip Banovac, Heather Bomberger, Joshua Jones, Peiman Habibollahi, Nariman Nezami

**Affiliations:** 1 Georgetown University School of Medicine, 3700 Reservoir Road NW, Washington, DC 20007 USA; 2 Department of Radiology, Emory University School of Medicine, 1364 Clifton Rd NE, Atlanta, GA 30322 USA; 3 Division of Interventional Radiology, MedStar Georgetown University Hospital, 3800 Reservoir Road NW, Washington, DC 20007 USA; 4 HIV and AIDS Malignancy Branch, Center for Cancer Research, NCI, Bethesda, MD USA; 5 Boston Scientific Corporation, 300 Boston Scientific Way, Marlborough, MA 01752 USA; 6 Department of Interventional Radiology, University of Texas MD Anderson Cancer Center, 1515 Holcombe Blvd, Houston, TX 77030 USA; 7 Lombardi Comprehensive Cancer Center, 3800 Reservoir Rd NW, Washington, DC 20007 USA; 8 Fischell Department of Bioengineering, University of Maryland College Park, 3102 A. James Clark Hall, College Park, MD 20742 USA

**Keywords:** Bleed, Embolization, Obsidio, Volume, Density

## Abstract

**Purpose:**

To characterize the temporal dynamic and clinical remodeling of the Obsidio™ Conformable Embolic (Obsidio) post endovascular delivery using longitudinal quantitative CT volumetry and Hounsfield unit (HU) attenuation.

**Materials and methods:**

This retrospective, single-center cohort study included 21 patients (30 vessels) embolized with Obsidio for acute hemorrhage or hypervascular tumor between 2023 and 2026. Patients had at least two follow-up computed tomography (CT) scans. The primary endpoint was the percent change in embolic cast volume and HU attenuation, measured using standardized Picture Archiving and Communication System (PACS) segmentation tools. Secondary endpoints included assessment for recanalization.

**Results:**

All embolizations were technically successful (100%). On the first follow-up CT (median 12 days), the mean Obsidio volume was 89.33 ± 13.86% of the original volume (*p* = 0.0002). On the second follow-up (median 36 days), the volume was 79.51 ± 19.76% of baseline volume (*p* = 0.0004), representing 20.49% reduction. On third follow-up CT (median 76 days), volume was 85.2 ± 18.92% (*p* = 0.047). On the fourth follow-up CT, volume was 66.76 ± 16.9% (*p* = 0.0048). On the fifth follow-up CT, volume was 60.15 ± 3.15% (*p* = 0.00001) The average volume reduction rate decelerated from 0.99%/day to 0.71%/day to 0.17%/day then stabilized at 0.2%/day. Concurrently, mean Hounsfield unit attenuation increased by 4.85% (*p* = 0.06) on the second follow-up CT scan. No evidence of recanalization, distal embolization, or cast fragmentation was observed on any of CT scans.

**Conclusion:**

The Obsidio embolic cast undergoes progressive reduction in volume and increase in HU attenuation over time without evidence of vessel recanalization. This temporal dynamic of Obsidio embolic is consistent with bioresorption of its gelatin and water components, supporting the concept of persistent occlusion via in vivo remodeling.

## Introduction

The management of active hemorrhage relies on a multimodal approach, encompassing mechanical, thermal, and chemical strategies to achieve hemostasis [1, 2]. In the realm of interventional radiology, transarterial embolization (TAE) is established as a primary image-guided modality for controlling hemorrhage, supported by Society of Interventional Radiology (SIR) guidelines [3]. Over time, embolization has advanced significantly, and today, interventionalists have access to a wide range of devices and agents. These include coils, plugs, and balloons to temporary and permanent particulate agents (non-calibrated and calibrated particles), liquids/gels (sclerosing agents, thrombin, in situ precipitating ethylene vinyl alcohol copolymer, and in situ polymerizing N-butyl-2-cyanoacrylate) [4].

The Obsidio™ Conformable Embolic (Obsidio; Boston Scientific) represents a novel addition to this toolkit. It is a pre-formed, shear-thinning hydrogel composed of bioresorbable gelatin, synthetic silicate nanoplatelets, tantalum, and water [5, 6]. Its cohesive, non-adhesive properties are designed for controlled delivery and conformable vessel filling. Early clinical reports have demonstrated a high rate of technical success for hemorrhage control and tumor embolization [7].

Obsidio’s evolution in vivo is unique, although it is incompletely characterized. As the gelatin matrix resorbs, the embolic cast is hypothesized to undergo volumetric reduction and an increase in Hounsfield unit (HU) attenuation while maintaining vessel occlusion. This imaging-based hypothesis is supported by preclinical data, where a Good Laboratory Practice (GLP) study in a porcine model demonstrated that Obsidio elicits a localized inflammatory response (macrophages, multinucleated giant cells) that breaks down the gelatin and silicate components by 90 days, leaving only tantalum aggregates integrated into a remodeled, permanently occluded lumen by 180 days [8]. This process is fundamentally distinct from in vivo reactions to other agents, such as coil compaction or liquid embolic recanalization. Standard follow-up imaging may reveal changes in the embolic cast that reflect natural material evolution. There remains a scarcity of quantitative, longitudinal imaging data detailing this process. Therefore, the purpose of this study was to quantify the temporal changes in Obsidio embolic cast volume and CT attenuation over time post-embolization period to define the expected imaging evolution of this material and distinguish normal remodeling from potential pathological findings. These findings are instrumental in optimizing the clinical use of Obsidio across a variety of medical indications.

## Materials and methods

### Study design and patient selection

This is a retrospective, single-center cohort study from Oct. 2023 till April 2026. All consecutive adult patients (≥ 18 years) who underwent TAE with Obsidio and had at least two post-procedural CT scans available for review after the index procedure were enrolled into this study. Indications for embolization were active, non-neurovascular hemorrhage or hypervascular tumor. Exclusion criteria were: life expectancy < 30 days, pregnancy, use of coil embolics along with Obsidio, embolization for indications outside the study scope (e.g., uterine fibroids, pulmonary AVMs), cancer-related bleeding from non-tumor sites, or a diagnosis of hematologic malignancy. Institutional review board approval was obtained with a waiver of informed consent. Uterine fibroid embolization was out of scope for this study because this is not a current indication for Obsidio. Pulmonary AVMs were excluded as Obsidio is contraindicated for use in pulmonary, coronary, and intracerebral vasculature. Additionally, uterine fibroid embolization and pulmonary AVM embolization involve distinct vascular beds where embolic cast behavior may differ meaningfully from the acute arterial hemorrhage setting.

### Embolization procedure

All procedures were performed via a transfemoral arterial approach. After diagnostic angiography identified the target vessel, a microcatheter was superselectively advanced. Obsidio is a pre-formed, ready-to-use, shear-thinning hydrogel requiring no mixing or preparation. The syringe was uncapped and connected directly to the microcatheter hub using a wet-to-wet technique to prevent air introduction and premature dehydration. Obsidio was injected under continuous fluoroscopic visualization until antegrade flow stasis was achieved, confirming technical success. The injection volume was determined by the operator using the clinical endpoint of antegrade flow stasis. The microcatheter was then removed without flushing to avoid displacing the embolic cast. No adjunctive embolic agents were used. Main catheter and sheath management followed standard institutional practice.

### Volumetric and HU attenuation analysis

All patients had CT imaging as part of standard clinical care. The initial post-procedural CT was typically obtained within 1–3 days. Follow-up CT scans (non-contrast and contrast-enhanced phases) were acquired based on clinical indication. For volumetric and densitometric analysis, non-contrast series were utilized to isolate the radiopaque tantalum signal of the Obsidio cast. Assessment for recanalization was performed using contrast-enhanced and angiographic phase images when available. When only a single phase was available, it was used for the analyses it permitted (e.g., non-contrast for volumetry; contrast-enhanced for recanalization assessment).

Quantification was performed using the volumetric segmentation tool within the Visage Picture Archiving and Communication System (PACS) platform (Visage Imaging). The embolic cast in each treated vessel was manually segmented on each available non-contrast CT by two independent readers. When the readers’ volume and HU attenuation measurements were concordant, the values were accepted. In cases of disagreement, the readers reviewed the segmentation together and reached a consensus. Each vessel was analyzed independently. The software calculated the total cast volume in mm^3^ and the mean HU attenuation within the segmented volume. Each vessel was analyzed independently.

**Fig. 1:**
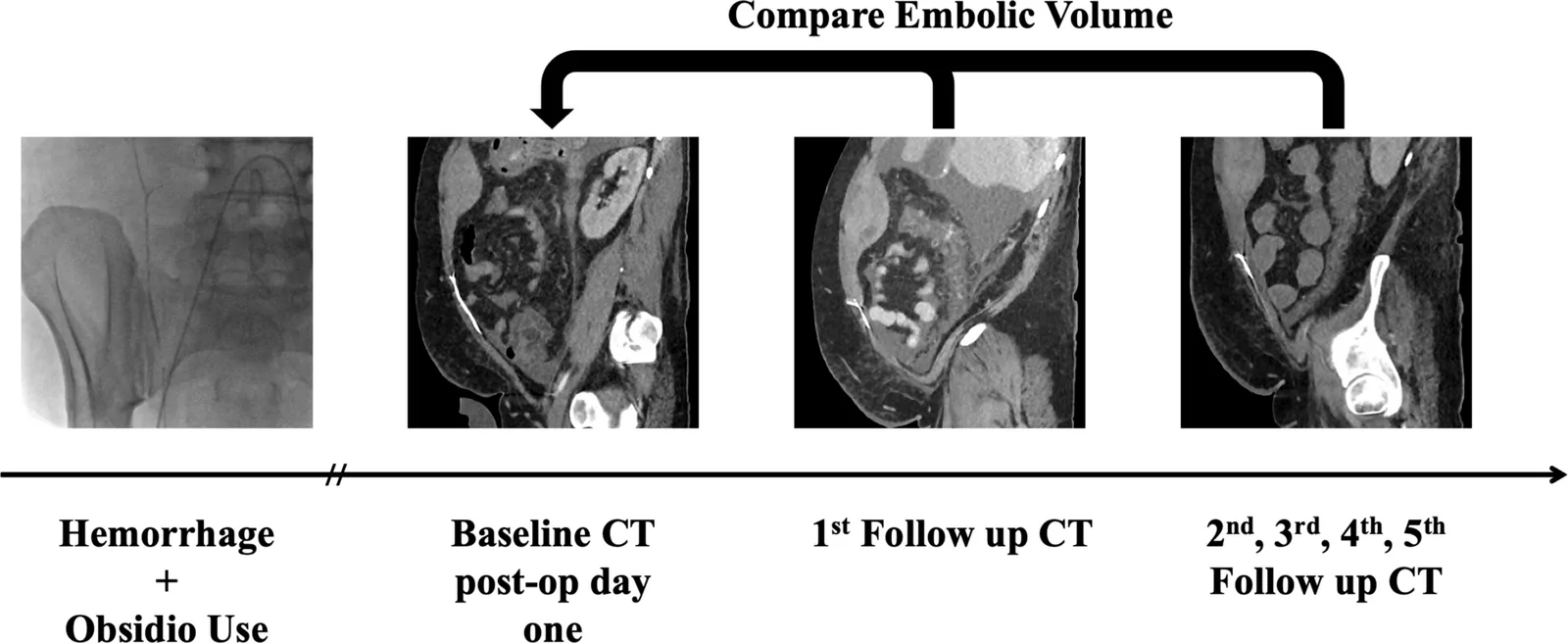
Study design schematic for longitudinal CT volumetry of Obsidio embolic casts

Figure 1 depicts the temporal sequence of the study. All patients underwent transarterial embolization using Obsidio. A baseline non-contrast CT scan was obtained post-procedurally to establish the initial cast volume (V_0_) and radiodensity (HU_0_). Patients then received at least one follow-up CT scan (first follow-up, V_1_, HU_1_). A subset of patients underwent a second follow-up CT (Second Follow-up, V_2_, HU_2_). A subset of patients underwent a third, fourth, or fifth follow-up. The primary outcome was the change in embolic volume calculated between V_0_ and V_1_/V_2_ and radiodensity between HU_0_ and HU_1_/HU_2_. Secondary endpoints included the rate of volume reduction (%/day) and assessment for recanalization.

### Outcome measures and statistical analysis

The primary endpoint was the change in Obsidio embolic cast volume and HU attenuation between the initial post-procedural CT and subsequent follow-up scans. Secondary endpoints included the rate of volume reduction (%/day) and the radiologic presence or absence of recanalization, defined as contrast opacification within or around the cast on angiographic phases.

Descriptive statistics were reported as mean ± standard deviation. A paired *t*-test was used to compare the embolic volume at baseline to the volume at each follow-up time point. Statistical significance was set at *p* < 0.05.

## Results

### Patient and procedural characteristics

Twenty-one patients (mean age 59 ± 17 years; 6 male and 15 female) met the inclusion criteria. Fifteen patients had a single artery embolized, three patients had two arteries embolized, and three patients had three arteries embolized, for a total of 30 vessels embolized. The treated vessels included visceral branches (gastroduodenal, gastric, adrenal, renal; *n* = 13), intercostal arteries (*n* = 11), and inferior epigastric/internal mammary arteries (*n* = 6). Treated vessels were embolized with 0.257 ± 0.069 mL of Obsidio, based on the interventionist intraoperative report. The indication was active hemorrhage in 25 vessels (83.3%) and a peripheral artery aneurysm/pseudoaneurysm in 5 vessels (16.7%). All procedures (100%) achieved technical success with immediate cessation of bleeding, including several trauma patients with associated coagulopathy in this study. The preprocedural INR ranged from 1 to 4.7 (mean 1.52 ± 0.84). Preprocedural hemoglobin ranged from 5.3 to 9.8 (mean 7 ± 1.1). Preprocedural platelets ranged from 53 to 314 (mean 191 ± 144).

### Volumetric analysis

**Fig. 2:**
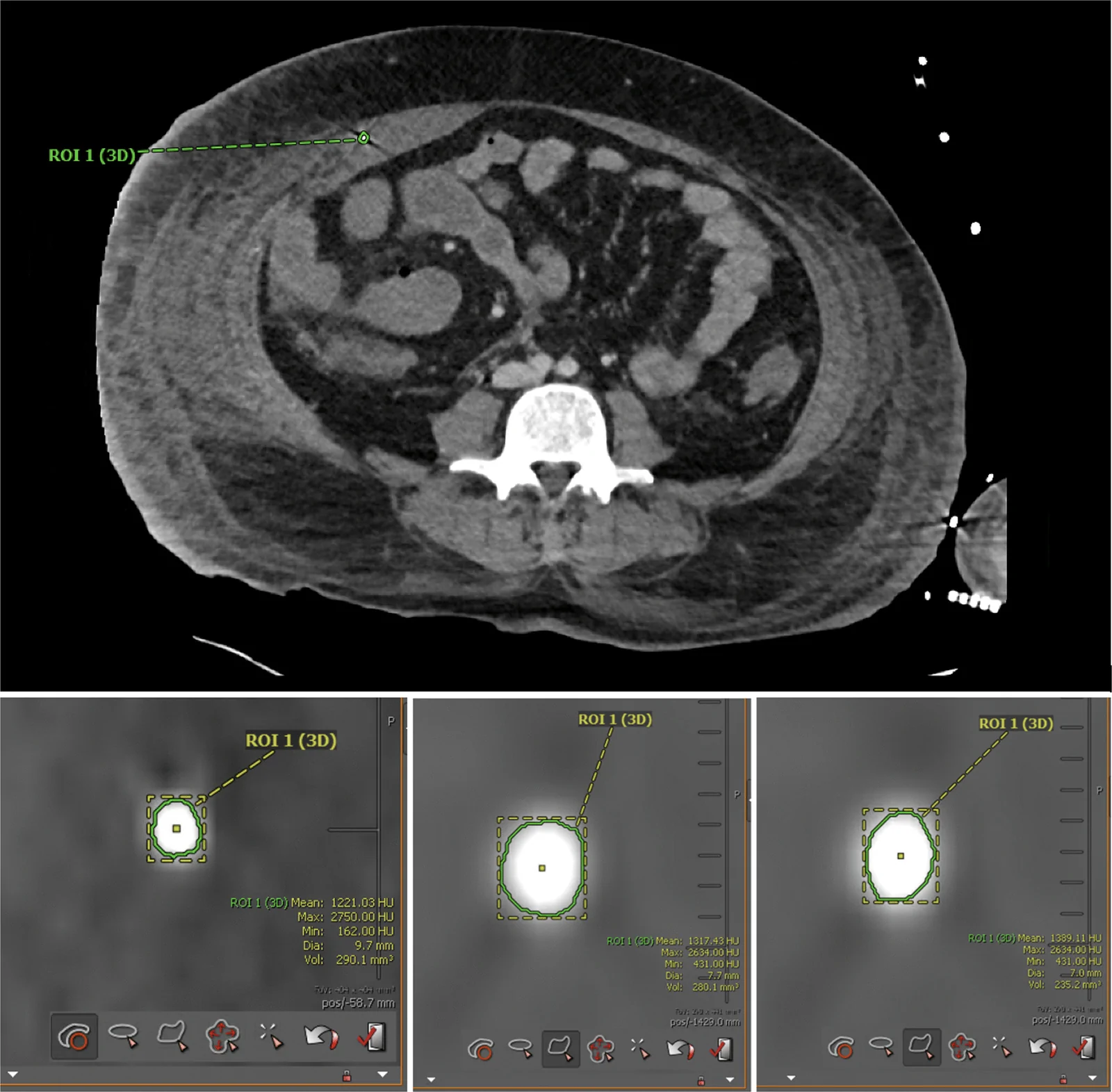
Serial axial CT images with volumetric segmentation (green overlay) illustrating the progressive reduction in volume of a cohesive Obsidio embolic cast over 32 days. The cast maintained morphological integrity without evidence of recanalization or fragmentation. Volumes: **A** 290.1 mm^3^ (baseline), **B** 280.1 mm^3^ (10-day follow-up), **C** 235.2 mm^3^ (32-day follow-up). This corresponds to a net volume loss of 18.9%

**Fig. 3:**
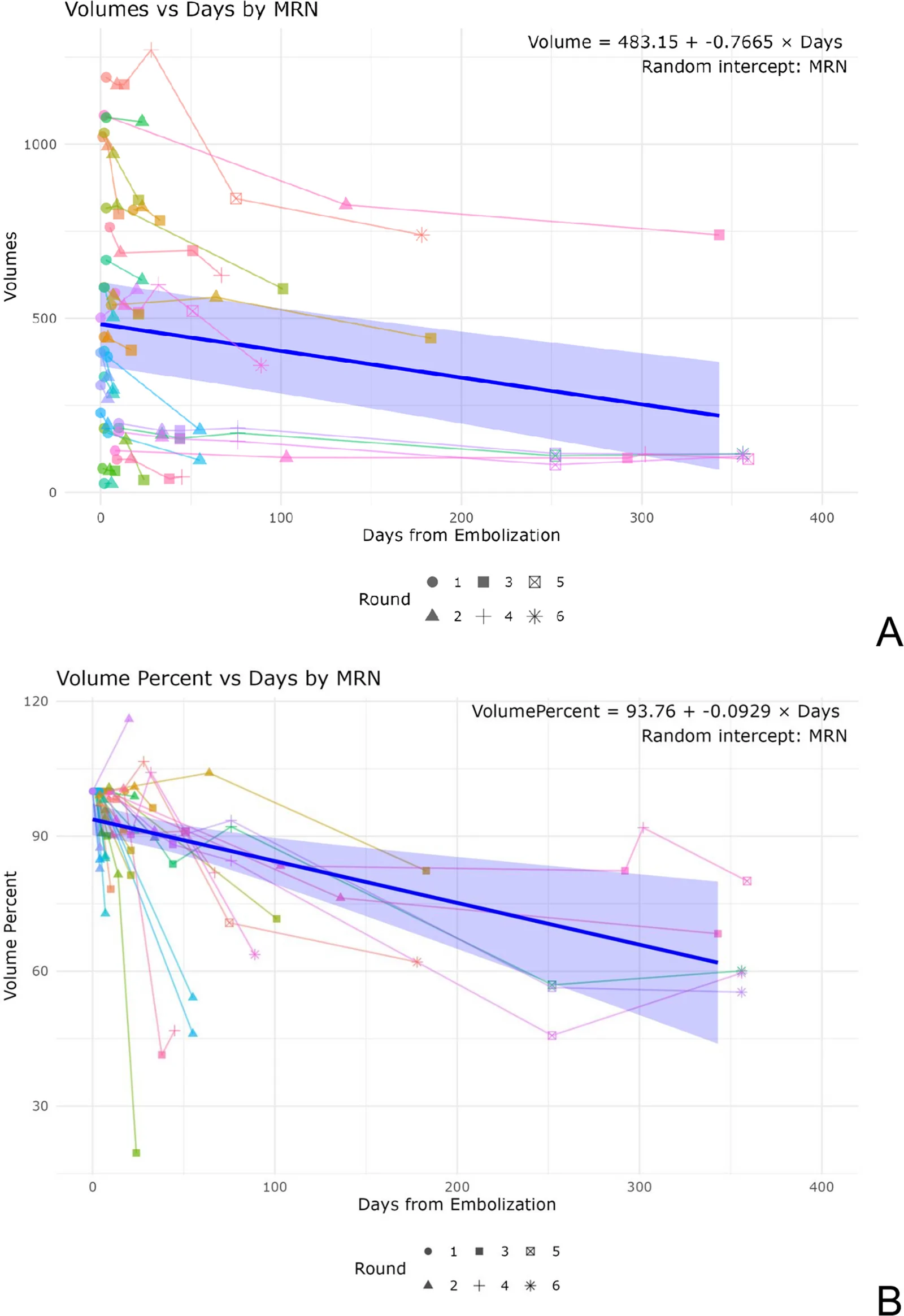
**A** Volume (in mm3) vs days from embolization: each patient is shown in a color. The blue line shows a random-effects linear model that controls for patient effects, with the ribbon showing the confidence interval. **B** Volume (in % relative to the first round) vs. Days from Embolization: Each patient is shown in a color. The blue line shows a random-effects linear model that controls for patient effects, with the ribbon showing the confidence interval

All patients had a first follow-up CT at a median of 12 days post-embolization. Fifteen patients (18 vessels) had a second follow-up CT at a median of 36 days. Seven patients (9 vessels) had a third follow-up CT at a median of 76 days. Four patients (6 vessels) had a fourth follow-up CT at a median of 252 days. Three patients (5 vessels) had a fifth follow-up CT at a median of 356 days. The quantitative change in volume is summarized in Table 1. At the first follow-up, the mean Obsidio volume was 89.33 ± 13.86% (95% CI: 84.16–94.51), of the original post-procedural volume, representing a significant 10.67% reduction (*p* = 0.0002). At the second follow-up, the mean volume further decreased to 79.51 ± 19.76% (95% CI: 69.68–89.34), of baseline, corresponding to a net 20.49% reduction from the initial volume (*p* = 0.0004). At the third follow-up, the mean volume was found to be 85.20 ± 18.92% (95% CI: 70.66–99.74), of baseline, corresponding to a net 14.8% reduction from the initial volume (*p* = 0.047). At the fourth follow-up, the mean volume decreased to 66.79 ± 16.89% (95% CI: 49.06–84.52), of baseline, corresponding to a net 33.21% reduction from the initial volume (*p* = 0.0048). At the fifth follow-up, the mean volume further decreased to 60.15 ± 3.15% (95% CI: 56.24–64.05), of baseline, corresponding to a net 39.85% reduction from the initial volume (*p* = 0.00001). The mean per-vessel rate of volume reduction decelerated and was then found to be stable over time, measuring 0.99%/day between the initial and first follow-up, and 0.71%/day between the initial and second follow-up, then 0.17%/day by the third follow-up, then 0.2%/day by the fourth follow-up, and 0.19%/day by the fifth follow-up. A representative case demonstrates the progressive volumetric reduction of the embolic cast over time, as shown in Fig. 2. The cast volume decreased from 290.1 mm^3^ post-procedurally to 280.1 mm^3^ at 10 days and 235.2 mm^3^ at 32 days, while maintaining a cohesive morphology without evidence of recanalization. The observed volumetric reduction over time is summarized in Fig. 3, which shows the decreasing trend in individual cast volumes from baseline to final follow-up.

**Table 1:** Longitudinal CT measurements of Obsidio embolic cast volume and Hounsfield unit (HU) attenuation

	**Baseline**	** 1 st follow-up and *P* value**	**2nd follow-up and *P* value**	**3rd follow-up and *P* value**	**4th follow-up and *P* value**	**5th follow-up and *P* value**
Preserved volume (%)	**100**	**89.33 *p* = 0.0002**	**79.51 *p* = 0.0004**	**85.20 *p* = 0.0469**	**66.79 *p* = 0.010**	**60.15 *p* = 0.00001**
Δ volume decrease/day (%/day)	**0**	**0.99**	**0.71**	**0.17**	**0.20**	**0.19**
HU increase (%)	**0**	**2.2 *p* = 0.4993**	**4.85 *p* = 0.0624**	**4.47 *p* = 0.3723**	** − 3.65 *p* = 0.3126**	** − 2.12 *p* = 0.7169**
Δ HU increase/day (%/day)	**0**	**0.55**	**0.11**	**0.08**	**−0.02**	**0.004**

Serial quantitative measurements from baseline post-procedural CT to 1 st, 2nd, 3rd, 4th, and 5th follow-up. Values are presented as mean. Changes and daily rates of change are calculated relative to baseline measurements. Statistical significance was determined using a paired *t*-test

^*^p-value < 0.05

### Radiodensity analysis

**Fig. 4:**
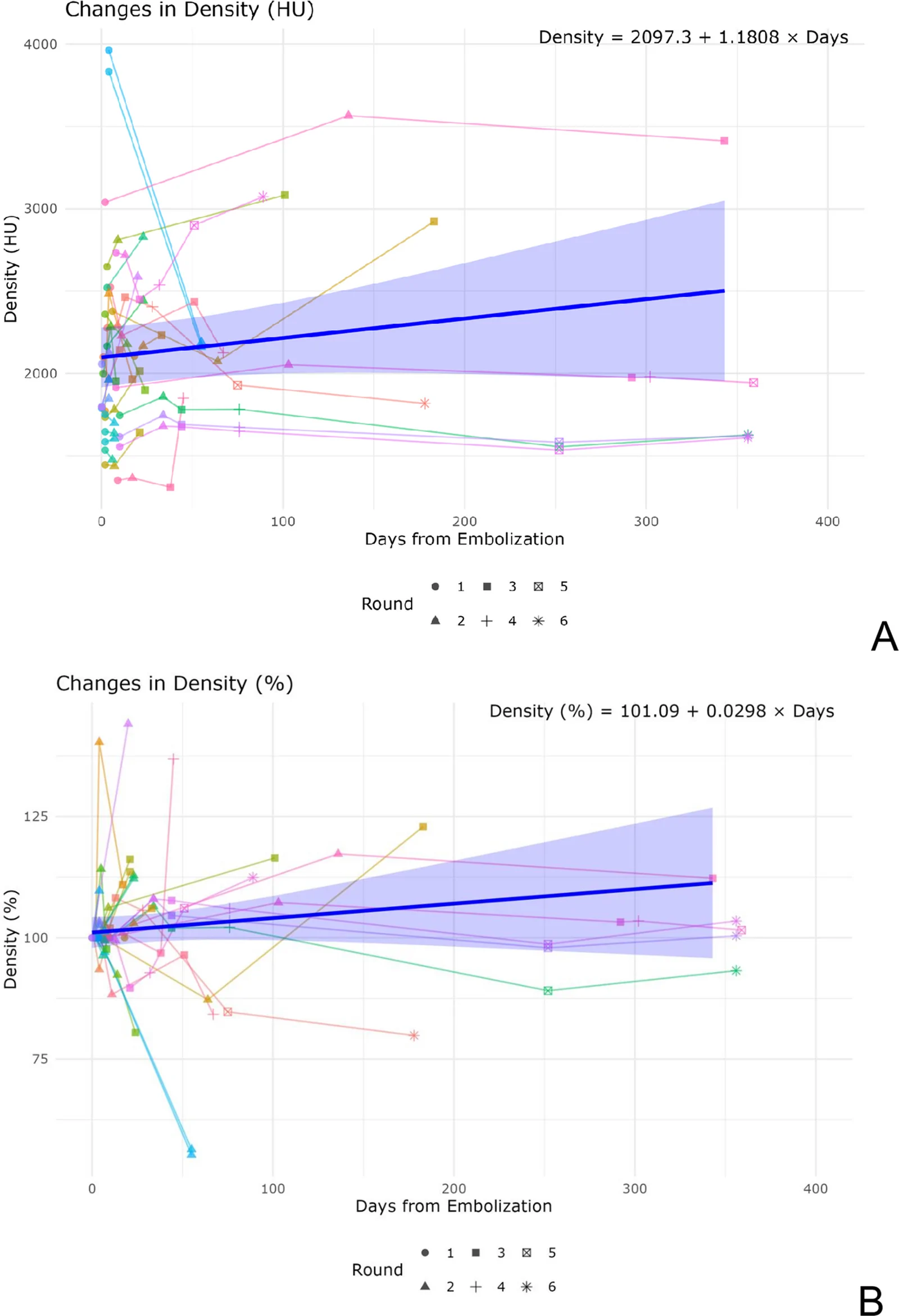
**A** Density (in HU) vs. days from embolization: each patient is shown in a color. The blue line shows a random-effects linear model that controls for patient effects, with the ribbon showing the confidence interval. **B** Density (in % relative to the first round) vs. days from embolization: each patient is shown in a color. The blue line shows a random-effects linear model that controls for patient effects, with the ribbon showing the confidence interval

Analysis of the embolic cast’s mean Hounsfield unit (HU) attenuation revealed a non-significant trend toward increasing HU attenuation at the first follow-up, with a mean change of + 2.2% (± 17.58%; *p* = 0.499), equivalent to an average mean per-vessel rate increase of 0.55% per day. At the second follow-up, this increase in attenuation had a mean change of + 4.85% (± 10.32%; *p* = 0.0624) from baseline, corresponding to a slower mean per-vessel rate of 0.11% per day. At the third follow-up, increase in attenuation had a mean change of + 4.47% (± 14.18%; *p* = 0.37) from baseline, corresponding to a slower mean per-vessel rate of 0.08% per day. At the fourth follow-up, decrease in attenuation had a mean change of − 3.65% (± 7.97%; *p* = 0.313) from baseline, corresponding to a mean per-vessel rate of − 0.02% per day. At the fifth follow-up, decrease in attenuation had a mean change of − 2.12% (± 12.2%; *p* = 0.7169) from baseline, corresponding to a mean per-vessel rate of 0.004% per day. To assess the general relationship between days from embolization and volume and density (in absolute terms or as a percentage relative to baseline CT), while controlling for patient effects, we use a random-effects model. To model the linear model and its confidence interval, we used R (v4.5.2) with packages lme4 (v2.0.6) and ggeffets (v2.3.2) in Figs. 3 and 4. Because the number of samples in follow-ups 4, 5, and 6 CT scans is limited, and these rounds may have outliers, we excluded them from training the random-effects model. The quantitative change in Hounsfield unit (HU) attenuation is summarized in Table 1.

### Assessment for recanalization

No evidence of recanalization, distal embolization, or cast fragmentation was observed on any follow-up CT angiogram. The morphology of the embolic material remained smooth and contiguous on all multiplanar reconstructions.

### Availability of data and materials

The datasets used and analyzed during the current study are available from the corresponding author on reasonable request.

## Discussion

This quantitative imaging study provides evidence that the Obsidio embolic cast undergoes a progressive, predictable reduction in volume and increase in radiodensity over time following endovascular delivery. This finding is consistent with the hypothesis of in vivo bioresorption of its gelatin and water components.

The observed volumetric decrease, approximately 10.67% from baseline to the first follow-up and 39.85% from baseline to the fifth follow-up, occurred without any radiologic evidence of vessel recanalization. This distinction is critical for clinical interpretation, as a shrinking cast on follow-up imaging may indicate a transition toward a more stable structure as non-resorbable components, such as tantalum, become the dominant elements of the material. The decelerating mean per-vessel rate of volume loss (from 0.99%/day to 0.19%/day) suggests decelerating gelatin hydrolysis and water diffusion, transitioning to a stable scaffold dominated by the non-resorbable tantalum components.

The concurrent measurements of volumetric reduction and increasing HU attenuation provide a more complete picture of the cast’s in vivo evolution. The non-significant trend increase in mean HU attenuation at the second follow-up is consistent with the bioresorption of the lower-attenuation gelatin matrix and the resultant relative concentration of the higher-attenuation tantalum component within the persistent cast [5, 6]. This behavior, a form of biomimetic remodeling, is supported by preclinical models showing that a localized inflammatory response degrades the gelatin and silicate components, leaving tantalum integrated into a permanently fibrosed lumen over 90–180 days [8]. The decelerating rate of HU attenuation increase (0.55% per day at 12 days versus 0.08% per day at 76 days) complements the rate of volumetric loss, suggesting the most rapid phase of gelatin resorption occurs within the first several weeks post-embolization. These correlated imaging metrics help differentiate active material remodeling from passive mechanical compression as the primary driver of cast volume loss, supporting the premise of a predictable biomimetic degradation process rather than uncontrolled fragmentation or washout.

The absence of recanalization in this cohort is a favorable outcome, indicating that the remodeling process does not compromise the primary goal of hemorrhage control. This stability may offer theoretical advantages over permanent implants that can compact or incite chronic inflammation. However, the long-term clinical implications of this slow remodeling, including its effect on permanent vessel fibrosis, warrant investigation. The primary residual inorganic component, tantalum, has an established biocompatibility profile from its long-term use in orthopedic and dental implants [9], which may mitigate concerns regarding its permanent retention.

The remodeling behavior of Obsidio differs from that of established embolic agents. Coils provide permanent mechanical occlusion without bioresorption but may compact over time, potentially leading to recanalization. Ethylene vinyl alcohol (EVOH) copolymer forms a stable, non-resorbable cast with minimal volume or attenuation change on follow-up imaging. Gelatin sponge undergoes rapid enzymatic degradation, often with vessel recanalization within days to weeks. Obsidio’s observed pattern of progressive volume reduction of approximately 39.85% over 356 days with an initial transient increase followed by a gradual decline in attenuation and no recanalization is consistent with gradual gelatin bioresorption and tantalum concentration, distinguishing it from both rapidly resolving temporary agents and permanently static liquid embolics.

### Limitations

This study has several limitations. Its retrospective design and reliance on clinically indicated imaging introduced variability in follow-up timing. All CT scans were obtained based on clinical necessity rather than a standardized research schedule, resulting in heterogeneous follow-up intervals across patients. While we reported mean follow-up times and calculated daily rates of volume and attenuation change to normalize for timing differences, prospective studies with predefined imaging time points would provide more precise characterization of the remodeling trajectory. This interval captures the early, active phase of gelatin bioresorption but is shorter than the 90- and 180-day time points reported in preclinical models, where complete gelatin resorption and chronic fibrotic integration were demonstrated. Whether late recanalization or further cast evolution occurs beyond the available scans cannot be assessed from our data and warrants investigation with longer-term prospective follow-up.

All CT examinations were performed according to institutional clinical protocols without study-specific standardization of acquisition parameters. Although all imaging was acquired at a single institution with relatively consistent protocols, inter-scan variation in tube voltage, current, and slice thickness could not be eliminated and may have introduced minor variability in Hounsfield unit measurements despite the use of patient-specific baseline normalization. Volumetric segmentation was performed by two independent readers with consensus resolution for discrepant cases. While this approach reduces individual reader bias, formal inter-observer and intra-observer reproducibility metrics were not calculated.

The small sample size (21 patients, 30 vessels), while sufficient to detect a statistically significant volumetric change, limits generalizability and precludes meaningful subgroup analysis. The cohort combined high-flow traumatic hemorrhage with five aneurysm/pseudoaneurysm cases, introducing pathophysiological heterogeneity that may influence remodeling kinetics. This study did not systematically account for clinical variables that may affect hemostatic outcomes, including peri-procedural anticoagulation management, reversal agents, or transfusion practices. CT volumetry, while objective for measuring tantalum-filled cast volume, cannot delineate the evolving tissue-cast interface or definitively distinguish between active bioresorption and passive compression; histopathological correlation was not available. Time from Obsidio injection to angiographic stasis was not recorded independently of total fluoroscopy time, and detailed peri-procedural coagulation parameters beyond exclusion thresholds were not systematically collected.

The third follow-up time point (median 76 days) demonstrated the greatest variability in volumetric measurements (85.20 ± 18.92%) and the lowest statistical significance (*p* = 0.047). This reflects both the smaller sample size at this interval (7 patients, 9 vessels) and the inherent heterogeneity of the remodeling process during the transition from active gelatin bioresorption to a more stable tantalum-dominant scaffold. Several individual vessels at this time point demonstrated apparent transient volume increases relative to the second follow-up, which may represent measurement variability, partial volume averaging, differences in segmentation boundaries, or true biological heterogeneity in resorption kinetics. Given the wide confidence interval (70.66–99.74%) and the non-monotonic trajectory observed between the second and third follow-ups, findings at this specific time point should be interpreted with caution. Larger prospective studies with standardized imaging schedules are needed to better characterize the remodeling dynamics during this intermediate phase.

Prospective studies with standardized imaging time points are needed to refine the understanding of this temporal evolution. Direct comparative studies against established embolic agents (e.g., coils, particles, EVOH) are essential to determine if the unique remodeling behavior of Obsidio translates into differences in patient-centered outcomes, such as rates of re-bleeding, post-embolization syndrome, or long-term vessel patency.

Additionally, while recanalization was assessed on contrast-enhanced and angiographic phases when available, some follow-up CT scans included only non-contrast acquisitions, which may limit the sensitivity for detecting partial recanalization.

## Conclusion

In conclusion, longitudinal CT volumetry and densitometry demonstrate that Obsidio undergoes a significant, predictable reduction in volume over time post-embolization, while concurrently increasing in HU attenuation and maintaining vascular occlusion without recanalization. This imaging profile supports the mechanism of in vivo biomimetic remodeling, characterized by the bioresorption of the gelatin matrix and stabilization and continued radiopacity by non-resorbable components. While these findings define the expected temporal evolution of the embolic cast, prospective and comparative research is necessary to establish whether this unique remodeling behavior translates into measurable long-term clinical advantages.

## Data Availability

The datasets generated and analyzed during the current study are not publicly available due to patient privacy regulations but may be available from the corresponding author on reasonable request and with appropriate institutional data use agreements.
